# Retinal inflammation in murine models of type 1 and type 2 diabetes with diabetic retinopathy

**DOI:** 10.1007/s00125-023-05995-4

**Published:** 2023-09-05

**Authors:** Subramanian Dharmarajan, Casandra Carrillo, Zhonghua Qi, Jonathan M. Wilson, Anthony J. Baucum, Christine M. Sorenson, Nader Sheibani, Teri L. Belecky-Adams

**Affiliations:** 1 Department of Biology, Indiana University—Purdue University Indianapolis, Indianapolis, IN USA; 2 Lilly Research Laboratories, Eli Lilly and Company, Indianapolis, IN USA; 3 Department of Pharmacology and Toxicology, Indiana University School of Medicine, Indianapolis, IN USA; 4 Department of Pediatrics, University of Wisconsin School of Medicine and Public Health, Madison, WI USA; 5 Department of Ophthalmology and Visual Sciences, University of Wisconsin School of Medicine and Public Health, Madison, WI USA; 6 Department of Cell and Regenerative Biology, University of Wisconsin School of Medicine and Public Health, Madison, WI USA

**Keywords:** Diabetic retinopathy, Gliosis, IFN gamma, IFNγ, Inflammation, PDGFRβ, Pericytes, PKCδ, Platelet-derived growth factor receptor β

## Abstract

**Aims/hypothesis:**

The loss of pericytes surrounding the retinal vasculature in early diabetic retinopathy underlies changes to the neurovascular unit that lead to more destructive forms of the disease. However, it is unclear which changes lead to loss of retinal pericytes. This study investigated the hypothesis that chronic increases in one or more inflammatory factors mitigate the signalling pathways needed for pericyte survival.

**Methods:**

Loss of pericytes and levels of inflammatory markers at the mRNA and protein levels were investigated in two genetic models of diabetes, *Ins2*^*Akita/+*^ (a model of type 1 diabetes) and *Lepr*^*db/db*^ (a model of type 2 diabetes), at early stages of diabetic retinopathy. In addition, changes that accompany gliosis and the retinal vasculature were determined. Finally, changes in retinal pericytes chronically incubated with vehicle or increasing amounts of IFNγ were investigated to determine the effects on pericyte survival. The numbers of pericytes, microglia, astrocytes and endothelial cells in retinal flatmounts were determined by immunofluorescence. Protein and mRNA levels of inflammatory factors were determined using multiplex ELISAs and quantitative reverse transcription PCR (qRT-PCR). The effects of IFNγ on the murine retinal pericyte survival-related platelet-derived growth factor receptor β (PDGFRβ) signalling pathway were investigated by western blot analysis. Finally, the levels of cell death-associated protein kinase C isoform delta (PKCδ) and cleaved caspase 3 (CC3) in pericytes were determined by western blot analysis and immunocytochemistry.

**Results:**

The essential findings of this study were that both type 1 and 2 diabetes were accompanied by a similar progression of retinal pericyte loss, as well as gliosis. However, inflammatory factor expression was dissimilar in the two models of diabetes, with peak expression occurring at different ages for each model. Retinal vascular changes were more severe in the type 2 diabetes model. Chronic incubation of murine retinal pericytes with IFNγ decreased PDGFRβ signalling and increased the levels of active PKCδ and CC3.

**Conclusions/interpretation:**

We conclude that retinal inflammation is involved in and sustains pericyte loss as diabetic retinopathy progresses. Moreover, IFNγ plays a critical role in reducing pericyte survival in the retina by reducing activation of the PDGFRβ signalling pathway and increasing PKCδ levels and pericyte apoptosis.

**Graphical Abstract:**

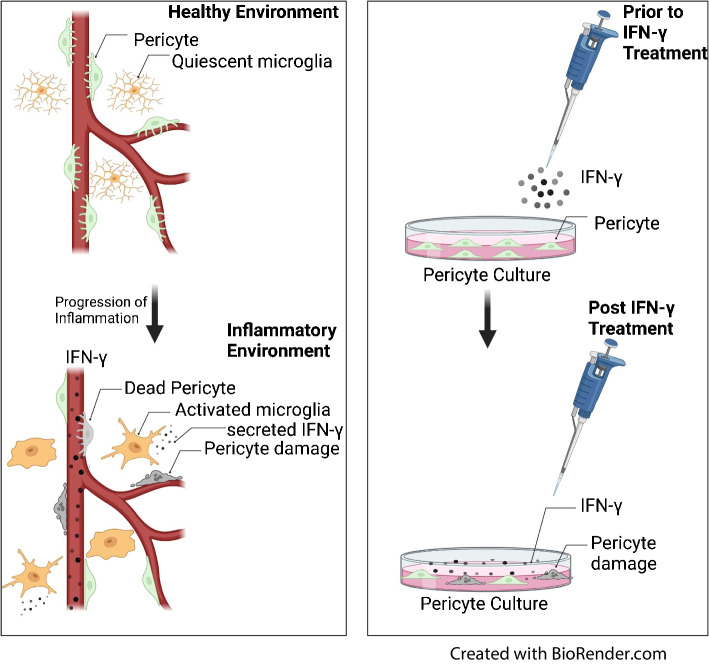

**Supplementary Information:**

The online version contains peer-reviewed but unedited supplementary material available at 10.1007/s00125-023-05995-4.


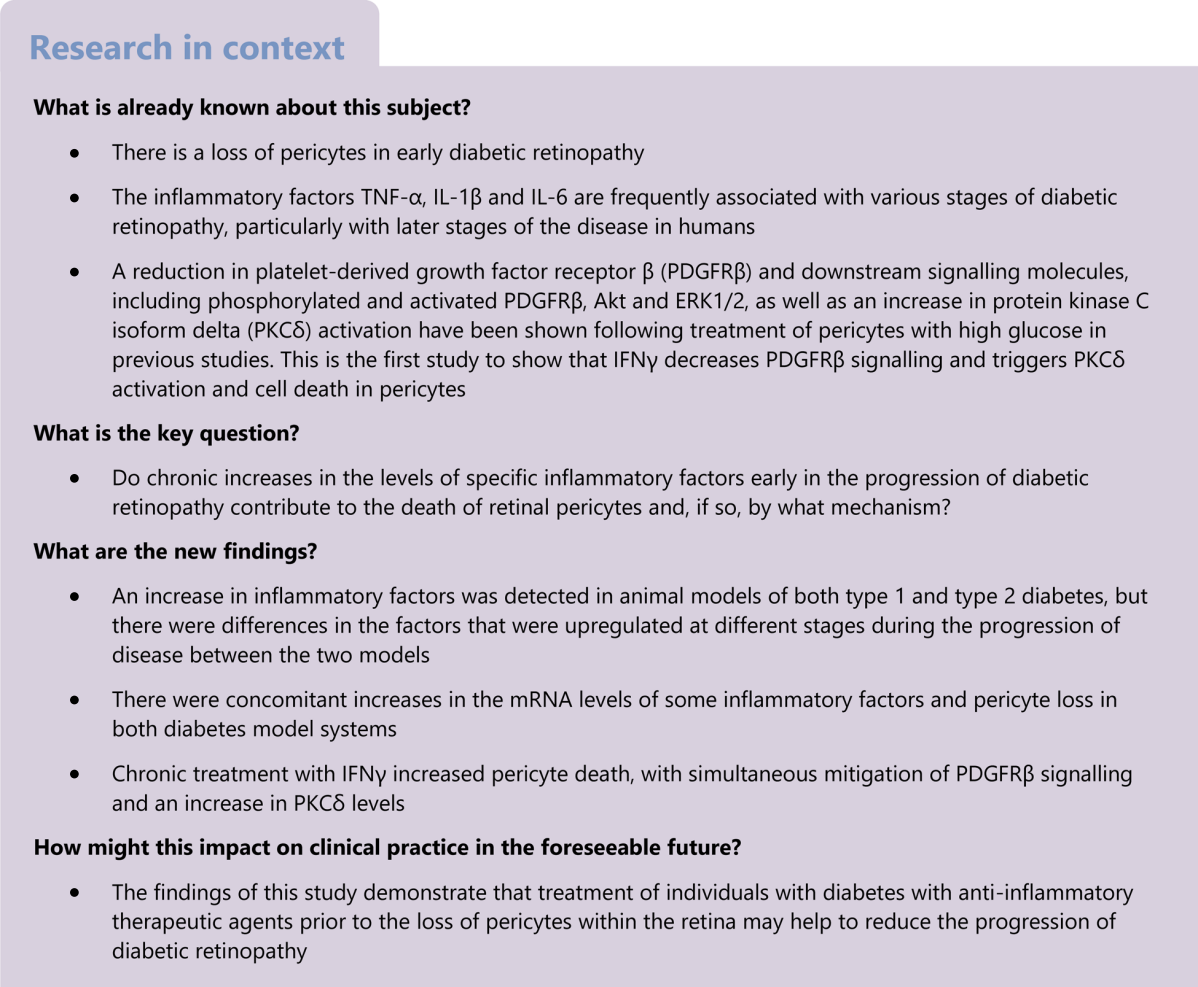


## Introduction

Diabetic retinopathy is one of the most common complications associated with both type 1 and type 2 diabetes, and is a leading cause of blindness worldwide [1]. Diabetic retinopathy is characterised as a disease of the retinal microvasculature, but it is accompanied by inflammation, oxidative stress, neuronal loss and gliosis [2]. There are two general forms of diabetic retinopathy. In order of their destructive influence, they are: (1) non-proliferative diabetic retinopathy, which may be further subdivided into mild, moderate and severe; and (2) proliferative diabetic retinopathy [3].

Pericytes, the contractile cells that cover the microvasculature, which is lined by endothelial cells, are one of the first cell types to be adversely affected by diabetes. A change in pericyte number is thought to underlie a cascade of early retinal vascular changes that occur during diabetes, including microaneurysms, thickening of the basement membrane, and loss of integrity of the blood–retina barrier and retinal neurons [4]. As the disease progresses, haemorrhaging, severe macular oedema, formation of hard exudates and cotton wool spots, continued neuronal loss, macro- and microgliosis, and neovascularisation occur [5]. Despite many valuable studies having been performed to try to understand the process by which the retinal pericytes are lost, mechanistic insight is lacking [4]. Both type 1 and type 2 diabetes involve hyperglycaemia, which leads to oxidative stress and the glycation of proteins and lipids [6]. Included among the glycated proteins is platelet-derived growth factor B (PDGFB), which is necessary for pericyte recruitment and survival [7]. While advanced glycated end-products (AGEs) lose their normal activity, they gain the ability to bind to the receptor for AGE (RAGE) or Toll-like receptor (TLR) 2 and/or 4 [8]. The RAGE and TLR pathways lead to increased transcription of proinflammatory genes [9]. Hyperglycaemia also increases the level of protein kinase C isoform delta (PKCδ), a member of the PKC family of kinases, involved in activating apoptosis of perivascular supporting cells [10]. While various inflammatory factors have been studied for their effects on the retinal vasculature, most of these studies were focused on later, more severe, stages in models of type 1 and type 2 diabetes [11]. Any potential role that inflammation may play in the early stages of the disease, particularly in pericyte loss, has not been clearly established.

To address the hypothesis that inflammation increases pericyte loss, we compared pericyte death, expression of inflammatory factors and glial activation in two genetic models of diabetes: *Ins2*^*Akita/+*^ (a model of type 1 diabetes) and *Lepr*^*db/db*^ (a model of type 2 diabetes). The role of acute and chronic IFNγ treatment in platelet-derived growth factor receptor β (PDGFRβ) signalling was subsequently tested in vitro using murine retinal pericytes.

## Methods

### Animals

All experiments described were performed in accordance with the Association for Research in Vision and Ophthalmology (ARVO) statement for the Use of Animals in Ophthalmic and Vision Research as well as guidelines issued by the institutions involved. Three lines of male mice were used for these studies: (1) the *Ins2*^*Akita*/+^ mouse, which carries a spontaneous mutation in the *insulin 2* gene on a C57Bl/6J background (C57BL/6-*Ins2*^*Akita*^/J; The Jackson Laboratory, USA; www.jax.org/strain/003548); (2) the *Lepr*^*db/db*^ mouse model of type 2 diabetes, which carries a mutation in the gene encoding the leptin receptor on a background of C57Bl/6J (B6.BKS(D)-*Lepr*^*db*^/J; The Jackson Laboratory; www.jax.org/strain/000697); and (3) the immortomouse line, which carries a temperature-sensitive simian virus 40 (SV40) large T antigen (CBA;B10-Tg(H2Kb-tsA58)6Kio/Crl; Charles River Laboratories, USA; www.criver.com/sites/default/files/resources/doc_a/rm_rm_d_immunodeficient_models.pdf). C57BL/6J mice (The Jackson Laboratory; www.jax.org/strain/000664) were used as wild-type (WT) mice. Only male mice were used for these studies due to limitations previously described in both *Ins2*^*Akita*/+^ and *Lepr*^*db/db*^ female mice [12, 13]. Specifically, both the *Ins2*^*Akita/+*^ and *Lepr*^*db/db*^ mouse lines have been shown to be sexually dimorphic; female *Lepr*^*db/db*^ mice have a severely reduced incidence of diabetes (only 12% of female *Lepr*^*db/db*^ mice are hyperglycaemic) [12] and *Ins2*^*Akita/*+^ male mice on multiple backgrounds have been shown to have ‘more robust’ hyperglycaemia in comparison with female mice [13]. Prior to being killed, weight and blood glucose readings were recorded for the animal models of diabetes. A non-fasting glucometer reading of 13.88 mmol/l was considered to indicate hyperglycaemia (Table 1); both *Ins2*^*Akita*/+^ and *Lepr*^*db/db*^ models were hyperglycaemic by 6 weeks of age. The PCR primers (*meta*bion, Munich, Germany) used to identify heterozygous *Ins2*^*Akita/+*^, homozygous *Lepr*^*db/db*^ and heterozygous immortomouse lines are given in Table 2. Animals were housed in an Association for Assessment and Accreditation of Laboratory Animal Care (AAALAC)-accredited facility in cages with four companions, and were kept on a 12 h light/dark cycle at 22°C with unrestricted access to standard mouse chow (LabDiet, USA) and water. Welfare assessments were performed daily. Animals were killed using CO_2_ and cervical dislocation.

**Table 1:** Blood glucose readings and weights from mice used for the study

Age of mice	WT		*Ins2*^*Akita*/+^		*Lepr*^*db/db*^	
	Blood glucose (mmol/l)	Weight (g)	Blood glucose (mmol/l)	Weight (g)	Blood glucose (mmol/l)	Weight (g)
3 weeks						
Mouse 1	4.8	11	5.3	10	5.6	14
Mouse 2	5.0	11	5.4	10	5.3	13
Mouse 3	5.3	10	5.6	10	6.5	13
Mouse 4	5.1	11				
6 weeks						
Mouse 1	6.2	22	19.9	14	20.5	34
Mouse 2	9.4	24	24.4	14	20.1	30
Mouse 3	9.8	20	29.2	16	15.1	32
Mouse 4	7.0	22	19.4	14	18.8	30
Mouse 5	8.7	22	25.2	16	14.9	28
Mouse 6	7.3	20	16.7	14	19.1	30
12 weeks						
Mouse 1	5.6	28	26.8	26	20.4	58
Mouse 2	6.3	26	16.5	24	15.8	56
Mouse 3	6.1	30	15.2	20	16.2	56
Mouse 4	6.1	30	27.3	28	17.6	56
Mouse 5	7.1	28	22.9	24	11.3	58
Mouse 6	7.1	30	24.1	26	19.9	58
18 weeks						
Mouse 1	8.3	30	15.4	24	26.5	62
Mouse 2	6.9	32	26.2	28	19.4	58
Mouse 3	7.2	28	21.9	28	19.6	60
Mouse 4	6.6	32	21.6	26	24.8	40
Mouse 5	7.0	30	25.3	28	30.8	44
Mouse 6	7.1	30	24.8	26	24.5	60
24 weeks						
Mouse 1	7.3	95	23.6	28	22.2	37
Mouse 2	7.2	100	22.2	25	24.2	38
Mouse 3	7.2	96	20.8	27	26.4	41
Mouse 4	7.2	96	23.2	22	24.8	42
Mouse 5	7.2	96	23.7	28	26.6	44
Mouse 6	7.3	98	23.0	26	24.3	42

**Table 2:** List of primers for qRT-PCR

Gene	Primer	Sequence (5ʹ→3ʹ)	Product length (no. of bases)
*Gfap*	Forward	TAGCCCTGGACATCGAGATCGCC	141
	Reverse	GGTGGCCTTCTGACACGGATTTGG	
*Vim*	Forward	AGGAAGCCGAAAGCACCCTGC	78
	Reverse	TCCGTTCAAGGTCAAGACGTGCC	
*Gs*	Forward	GCGCTGCAAGACCCGTACCC	145
	Reverse	GGGGTCTCGAAACATGGCAACAGG	
*Egfr*	Forward	ACCTATGCCACGCCAACTGTACCT	82
	Reverse	TGAACGTACCCAGATGGCCACACTT	
*Tlr4*	Forward	TGCCTGACACCAGGAAGCTTGA	102
	Reverse	AGGAATGTCATCAGGGACTTTGCTG	
*Pcan*	Forward	ATCCCTGAGTGGGGAAGGCACA	96
	Reverse	AGCAGGGGATGCTGGGTGATGA	
*Ncan*	Forward	CCTGACAAGCGTCCATTCGCCA	90
	Reverse	ACTGTCCGGTCATTCAGGCCGAT	
*S100b*	Forward	GACTGCGCCAAGCCCACACC	142
	Reverse	TCCAGCTCGGACATCCCGGG	
*Timp2*	Forward	GCAACAGGCGTTTTGCAATG	71
	Reverse	CGGAATCCACCTCCTTCTCG	
*Mmp14*	Forward	TGGGCCCAAGGCAGCAACTT	89
	Reverse	CGTTGTGTGTGGGTACGCAGGT	
*Gm-csf*	Forward	AGTCGTCTCTAACGAGTTCTCC	178
	Reverse	AACTTGTGTTTCACAGTCCGTT	
*Csf*	Forward	ACCAAGAACTGCAACAACAGC	91
	Reverse	GGGTGGCTTTAGGGTACAGG	
*Ifnα*	Forward	CAAGCCATCCCTGTCCTGAG	131
	Reverse	TCATTGAGCTGCTGGTGGAG	
*Ifnγ*	Forward	CAACAGCAAGGCGAAAAAGGA	90
	Reverse	AGCTCATTGAATGCTTGGCG	
*Il1β*	Forward	TGTCTGAAGCAGCTATGGCAA	141
	Reverse	GACAGCCCAGGTCAAAGGTT	
*Il6*	Forward	ACTTCACAAGTCGGAGGCTT	111
	Reverse	TGCAAGTGCATCATCGTTGT	
*Vegf*	Forward	ACTGGACCCTGGCTTTACTG	74
	Reverse	CTCTCCTTCTGTCGTGGGTG	
*Ccl5*	Forward	TGCCCACGTCAAGGAGTATTT	111
	Reverse	ACCCACTTCTTCTCTGGGTTG	
*Thbs1*	Forward	GCCACAGTTCCTGATGGTGA	149
	Reverse	TTGAGGCTGTCACAGGAACG	
*Thbs2*	Forward	GGGAGGACTCAGACCTGGAT	105
	Reverse	CGGAATTTGGCAGTTTGGGG	
*Cd68*	Forward	AAGGGGGCTCTTGGGAACTA	139
	Reverse	AAGCCCTCTTTAAGCCCCAC	
*Iba1*	Forward	ACGAACCCTCTGATGTGGTC	118
	Reverse	TGAGGAGGACTGGCTGACTT	
*Irf8*	Forward	CGGATATGCCGCCTATGACA	73
	Reverse	CTTGCCCCCGTAGTAGAAGC	
*B2m*^a^	Forward	AGATGAGTATGCCTGCCGTG	120
	Reverse	TCATCCAATCCAAATGCGGC	
*Sdha*^a^	Forward	GGACAGGCCACTCACTCTTAC	130
	Reverse	CACAGTGCAATGACACCACG	
*Srp14*^a^	Forward	CCTCGAGCCCGCAGAAAA	134
	Reverse	CGTCCATGTTGGCTCTCAGT	

^a^Used as a reference gene

### Immunofluorescence and quantitative reverse transcription PCR

**Fig. 1:**
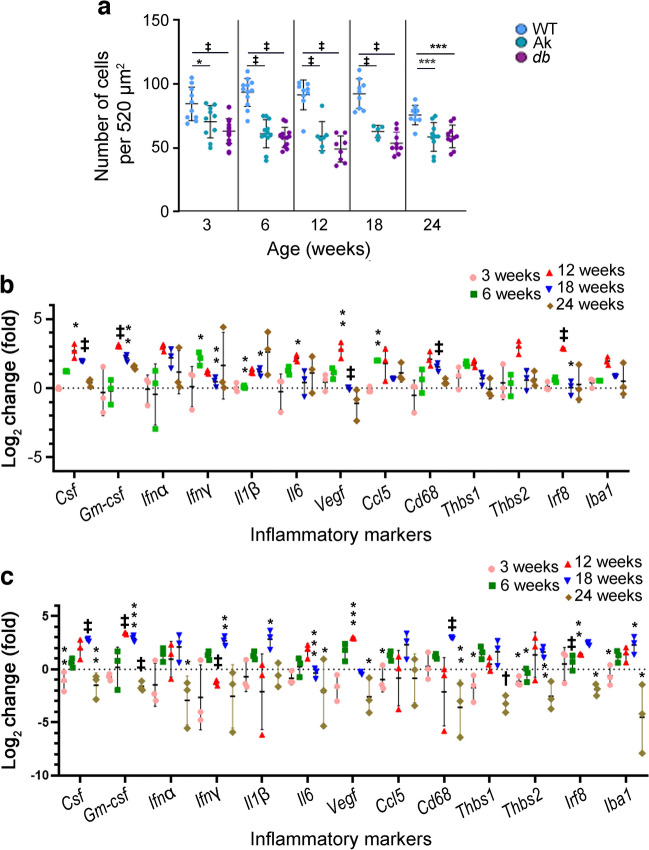
Pericyte number and inflammation levels are altered in the retinas of *Ins2*^Akita/+^ (Ak) and *Lepr*^*db/db*^ (*db*) mice. (**a**) Murine retinal flatmounts from 3-, 6-, 12-, 18- and 24-week-old WT, Ak and *db* mice were immunolabelled with the pericyte marker NG2 and the number of positive cells per 520 μm^2^ of tissue was quantified. (**b**, **c**) Quantification of qRT-PCR data showing the log_2_ fold change in mRNA for inflammatory factors in Ak mice (**b**) and *db* mice (**c**) relative to WT levels at 3, 6, 12, 18 and 24 weeks of age. WT levels were set to a value of 1.0, whereas the dotted line indicates a value of 0. Statistical analysis of RNA levels for each marker were compared for WT vs Ak (**b**) or WT vs *db* (**c**) across all ages. Overall, these figures show that pericyte loss was present at 3 weeks of age, with the level of loss plateauing at 12 weeks of age in Ak and *db* models. Detectable increases in proinflammatory mRNA levels occurred at 6 weeks of age in Ak retinas, but then peaked at 12 weeks and decreased at 18 weeks and 24 weeks of age in comparison vs WT retinas. In comparison, levels of many proinflammatory mRNAs decreased at 3 weeks and 24 weeks of age in retinas from *db* mice, then increased at 12 weeks and plateaued at 18 weeks of age compared with WT. Data are presented as mean±SEM; for (**a**), *n*=5–6 biological replicates, with six technical replicates per retina; for (**b**) and (**c**), *n*=3 biological replicates, with three technical replicates.*p<0.05, **p<0.01, ***p<0.001, ^‡^*p*<0.0001, analysed by one-way ANOVA with Tukey post hoc analysis

Information concerning primary antibodies and concentrations used are provided in Table 3. All antibodies were used as per manufacturers’ instructions. Retinal flatmount immunohistochemistry and analysis were performed as previously described [14]. Briefly, enucleated eyes were washed with 1× PBS, fixed in 4% paraformaldehyde (wt/vol.) at 4°C, rinsed with PBS and then stored at −20°C in methanol until used. Digital images were captured for 3–5 regions at a distance of 1–1000 µm away from the optic disc and >1000 µm away from the optic disc, and images were analysed using Fiji (2016; https://fiji.sc/) or Angiotool software (2016; https://ccrod.cancer.gov/confluence/display/ROB2/Downloads) to determine cell numbers (see electronic supplementary material [ESM] Fig. 1) [15]. Immunocytochemistry was performed as previously described [16]. For immunocytochemistry, cells were visualised using a Nikon Eclipse TE2000-U inverted microscope (Nikon Corporation, Japan). Primary antibodies were diluted in 2% serum (vol./vol.) diluted in 1× PBS and secondary antibodies were diluted in 0.3% Triton X (vol./vol.) in 1× PBS (PBST). The number of labelled cells in 20 fields was manually counted and divided by the total number of cells labelled with Hoechst stain to obtain the percentage of labelled cells. Quantitative reverse transcription PCR (qRT-PCR) was performed as previously described [14, 16]. The geometric means of the crossing threshold (C_t_) values of three housekeeping genes, *B2m*, *Sdha* and *Srp14*, were used as endogenous controls to normalise expression between samples. Primers used for PCR are listed in Table 2.

**Table 3:** Antibodies/lectin used in western blotting and immunohistochemistry

Antibody/lectin	Antibody dilution			Catalogue no.	Vendor	RRID no.
	Western blotting		IHC			
Antibody						
p-PDGFRβ (Tyr751)	1:1000		NA	MAB9027	R&D Systems	AB_2162777
PDGFRβ	1:1000		NA	3169	Cell Signaling Technology	AB_2162497
p-p44/42 MAPK (Thr202/Tyr204)	1:1000		NA	9101	Cell Signaling Technology	AB_331646
P44/42 MAPK	1:1000		NA	9102	Cell Signaling Technology	AB_330744
p-Akt (Ser473)	1:1000		NA	4060	Cell Signaling Technology	AB_2315049
Akt	1:1000		NA	2920	Cell Signaling Technology	AB_1147620
PKCδ	1:1000		NA	Gtx50605	Gene Tex	AB_11162869
β-Tubulin	1:1000		NA	T0198	Sigma	AB_477556
Peroxidase-conjugated goat anti-rabbit	1:4000		NA	32460	Thermo Fisher Scientific	AB_1185567
Peroxidase-conjugated goat anti-mouse	1:4000		NA	32430	Thermo Fischer Scientific	AB_1185566
Goat anti-rabbit IgG, HRP-linked	1:8000		NA	7074	Cell Signaling Technology	AB_20999233
Horse anti-mouse IgG, HRP-linked	1:8000		NA	7076	Cell Signaling Technology	AB_330924
Glial fibrillary acidic protein	NA		1:150	Z0334	Dako	AB 10013382
SOX2	NA		1:150	SC17320	Santa Cruz	AB 22686684
IBA1	NA		1:150	019-19741	Wako	AB 839504
NG2	NA		1:200	05-710	Millipore	AB 309925
CC3	NA		1:100	AF835	R&D Systems	AB 2243952
PCNA	NA		1:200	2586	Cell Signaling Technology	AB 2160343
Alexa Fluor	NA		1:500	A-11034, A-11055	Thermo Fisher Scientific	AB 2576217, AB 142672
Lectin						
IB4	NA	1:300		I21411	Thermo Fisher Scientific	AB 2314662

Manufacturer details for manufacturers not given in main text: Cell Signaling Technology, Danvers, MA, USA; Gene Tex, Irvine, CA, USA; Sigma, St Louis, MO, USA; Dako, Lexington, MA, USA; Santa Cruz, Dallas, TX, USA; Wako, Richmond, VA, USA; Millipore, Burlington, MA, USA

HRP, horseradish peroxidase; IHC, immunohistochemistry; MAPK, mitogen-activated protein kinase; NA, not applicable; RRID, research resource identifier

### Retinal pericyte isolation and culture

Mycoplasma-free murine retinal pericytes were isolated from the immortomouse line and grown as described previously [17]. Cells were maintained at 33°C and passaged every 3 days using 0.25% trypsin-EDTA. For assays testing the effects of IFNγ, cells were grown at 37°C, which is non-permissive for the expression of the SV40 large T antigen. For some experiments, cells were incubated with 1µl of vehicle (4 mmol/l HCl), 1µl PBS, 1 ng, 25 ng, 50 ng or 100 ng mouse IFNγ, and/or 50 ng/ml of recombinant human platelet-derived growth factor B homodimer (PDGFBB) (R&D Systems, MN, USA). Cultures treated with high glucose and osmotic controls (5.7 mmol/l d-glucose + 35 mmol/l l-glucose) and/or IFNγ were grown for 5 days, as described previously [18].

### Immunoblotting

Western blots were performed as previously described with minor modifications in the Revert Total Protein Stain and antigen pretreatment procedures [16]. All antibodies were used as per manufacturers’ instructions. Specifically, membranes were blocked with either 5% milk (wt/vol.) in TBS Tween 20 (TBST; 20 mmol/l Tris base pH 7.6, 137 mmol/l sodium chloride, 0.1% Tween 20 [vol./vol.]) or 10% BSA in TBST, for 1 h at room temperature. After blocking, membranes were incubated with primary antibody diluted in TBST overnight, at 4°C, on a shaker. Blots were then washed three times for 10 min and once for 5 min in TBST, incubated with SuperSignal West Femto or SuperSignal West Pico chemiluminescent substrate (Thermo Fisher Scientific, USA), and visualised on autoradiography film (Bio Dot Blue Lite, Dot Scientific, Burton, MI, USA). β-Tubulin was used as a loading control for the majority of blots, except for PKCδ immunoblots, whereby Revert Total Protein Stain (LI-COR, Lincoln, NE, USA) was used as a loading control, as per manufacturer’s instructions. Membranes probed for p-PDGFRβ were washed three times with ultrapure water and incubated with the antigen pretreatment solution in the SuperSignal Western Blot Enhancer kit (Thermo Fisher Scientific; catalogue no. 46641) for 10 min at room temperature, before washing five times with ultrapure water prior to the blocking step. In addition, membranes probed for p-PDGFRβ were incubated with the antigen pretreatment solution in a western blot enhancer signal kit (Thermo Fisher Scientific; catalogue no. 46641) for 10 min at room temperature prior to the blocking step.

### Multiplex ELISA

Levels of cytokines were measured from retinal samples using V-PLEX Proinflammatory Panel 1 ELISA Meso Scale Discovery (MSD) electrochemiluminescence kits (Meso Scale Diagnostics, Rockville, MD, USA; catalogue no. K15048D-1). Retinal samples from 6- and 12-week-old WT), *Ins2*^*Akita*/+^ and *Lepr*^*db/db*^ mice were dissected free of other tissues, flash-frozen in liquid nitrogen, and kept at −80°C prior to protein extraction and ELISA. Both retinas from the same mouse were pooled together to obtain enough protein to run the ELISA assays.

### Study design and statistics

**Fig. 2:**
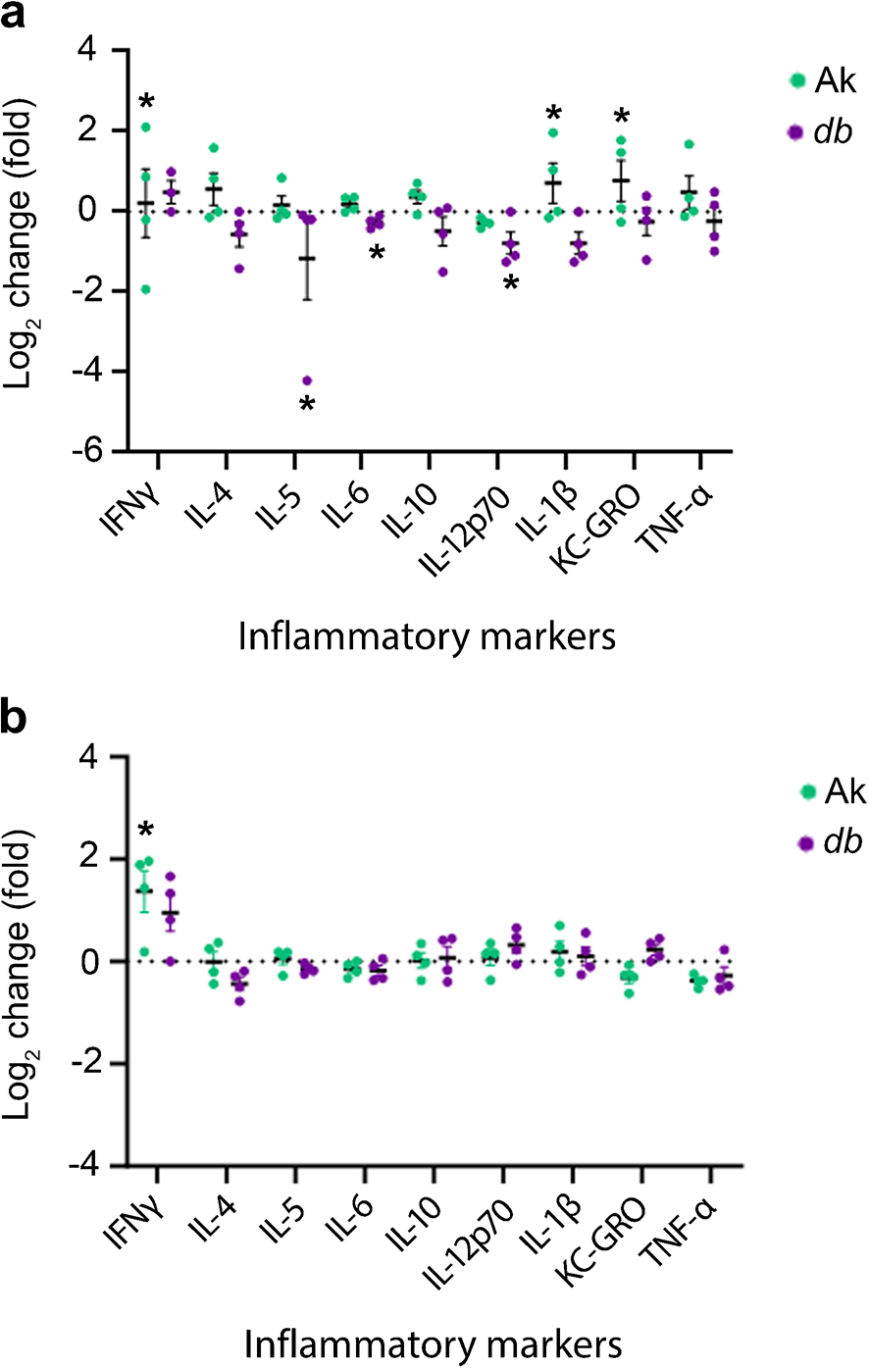
Proinflammatory markers in *Ins2*^*Akita/+*^ (Ak) and *Lepr*^*db/db*^ (*db*) mice at 6 and 12 weeks of age. (**a**, **b**) Levels of proinflammatory proteins in whole retinal samples were examined in samples from WT, Ak and *db* mice, obtained at 6 weeks (**a**) and 12 weeks (**b**) of age using multiplex ELISA panels. WT levels were set to a value of 1.0, whereas the dotted line indicates a value of 0. At 6 weeks of age, retinas from Ak mice showed an increase in IFNγ, IL-1β and keratinocyte chemoattractant/growth-related oncogene (KC-GRO) in comparison with WT, whereas retinas from *db* mice showed decreases in IL-5, IL-6 and IL-12p70. At 12 weeks, IFNγ was the only factor that increased in Ak mice compared with WT. Data are presented as mean±SEM; *n*=4 biological replicates. *p<0.05, analysed by one-way ANOVA. One outlier in the 6-week-old *db* mice data was identified using Grubbs’ analysis and was omitted from the analysis and graph

Samples used for quantification of retinal flatmounts, ELISAs and western blots were not randomised and the investigator was not blinded. In vitro samples were not randomised but the investigator was blind to the condition of the cells counted. Cell count data, qRT-PCR data and western blot densitometry data were analysed by one-way ANOVA [19] with Tukey’s test for post hoc analysis using Prism 9 (Prism 9.4.1; GraphPad Software, USA). Grubbs’ analysis was routinely run on all cell count, qPCR, western blot and ELISA data. Grubbs’ analysis identified an outlier in ELISA-derived data for proinflammatory proteins in whole retinal samples (Fig. 2) using GraphPad Prism. A *p* value ≤0.05 was considered statistically significant. Immunoassay data were fitted to an ANOVA model, with genotype, time (week) and their interaction as model terms. Comparisons were made between different genotypes at the same time points, or over time for the same genotype. The *p* values were not adjusted for multiple testing. Data are expressed as means with SEM, as indicated in the figure legends.

## Results

### Pericyte loss was evident at 3 weeks of age in retina of ***Ins2***^***Akita***/+^ and ***Lepr***^***db/db***^ models

Pericyte loss is an early marker of diabetic retinopathy in models of diabetes; however, a quantitative analysis of earlier timepoints in genetic models is lacking [20]. Early stages of the disease were designated as 12 and 18 weeks of age, and 24 weeks of age was designated as an intermediate stage, based on features such as pericyte loss, basement membrane thickening and changes to blood flow as described in other studies [21]. In this study, we refer to the age of the mice rather than the number of weeks the animals have had diabetes; however, Table 4 shows the conversion of age to number of weeks with diabetes for each genotype. *Ins2*^*Akita*/+^ mice become hyperglycaemic at 4 weeks of age. All of the *Lepr*^*db/db*^ mice in our study were hyperglycaemic by 6 weeks of age, although The Jackson Laboratory indicates that the mice can become hyperglycaemic between 4 and 8 weeks of age. Pericyte loss in WT mice, and models of type 1 and type 2 diabetes was compared using the *Ins2*^*Akita*/+^ and *Lepr*^*db/db*^ models. To quantify retinal pericytes in the early progression of the disease, retinas were obtained from *Ins2*^*Akita*/+^ and *Lepr*^*db/db*^ mice at 3, 6, 12, 18 and 24 weeks, and retinal flatmounts were immunolabelled for neural-glial antigen 2 (NG2; Fig. 1a and ESM Fig. 2). Quantification of these samples indicated a loss of pericytes as early as 3 weeks of age and continuing up to 12 weeks (Fig. 1a). In non-diabetic retinas, the mean number of NG2^+^ cells per 520 μm^2^ was 85±4, whereas in diabetic retina from 3-week-old *Ins2*^*Akita*/+^ and *Lepr*^*db/db*^ mice, the number of NG2^+^ cells was reduced to 71 (±4) and 63 (±2), respectively. At 6 weeks, the mean number of NG2^+^ cells increased in the WT to 94±3, but continued to decrease in the *Ins2*^*Akita*/+^ (61±3) and *Lepr*^*db/db*^ (58±2) mice. The number of pericytes continued to decrease, with the lowest numbers being observed at 12 weeks of age in both the *Ins2*^*Akita*/+^ mice (59±4) and *Lepr*^*db/db*^ (49±4) mice vs non-diabetic levels (92±4). At 18 and 24 weeks of age, levels of NG2^+^ cells in samples from *Ins2*^*Akita*/+^ (62±4.7 and 58±4, respectively) and *Lepr*^*db/db*^ (54±3 and 59±4, respectively) mice were not statistically different from 12 week samples. When compared with changes in blood glucose levels (Table 1), the number of pericytes began to decrease prior to the mice becoming notably hyperglycaemic.

**Table 4:** Conversion of age of mice to weeks with diabetes

	Time with diabetes (weeks)	
Age (weeks)	*Ins2*^*Akita*/+^	*Lepr*^*db/db*^
3		
6	2	
12	8	6
18	14	12
24	20	18

### Proinflammatory markers increased in retinas of ***Ins2***^***Akita***/+^ and ***Lepr***^***db/db***^ mice at early stages of diabetes

To determine the time course of retinal inflammation in the various mouse lines, RNA was isolated from WT, *Ins2*^*Akita*/+^ and *Lepr*^*db/db*^ retinas at 3, 6, 12, 18 and 24 weeks of age and the samples were subjected to qRT-PCR to analyse mRNA levels of secreted proinflammatory cytokines and chemokines that are known to change in those with diabetes, including colony-stimulating factor (encoded by *Csf1* [also known as *Csf*]), granulocyte macrophage colony-stimulating factor (encoded by *Csf2* [also known as *Gm-csf*]), IFNα (*Ifna* [*Ifnα*]), IFNγ (*Ifng* [*Ifnγ*]), IL-1β (*Il1b* [*Il1β*]), IL-6 (*Il6*), vascular endothelial growth factor (*Vegfa* [*Vegf*]), C–C motif chemokine ligand 5 (*Ccl5*) and thrombospondins 1 and 2 (*Thbs1* and *Thbs2*) [11, 22]. In addition, the levels of two markers that respond to proinflammatory signalling, *Irf8* and *Aif1* (also known as *Iba1*), were also determined [23, 24]. In retinas of the *Ins2*^*Akita*/+^ mice, there were no statistically significant increases in inflammatory markers at 3 weeks of age relative to control littermates (Fig. 1b). In contrast, by 6 weeks of age, there were significant increases in the levels of *Ifnγ* and *Ccl5* in comparison with WT littermates. The peak increase in mRNA levels occurred for many of the markers at 12 weeks of age, with increases in *Csf*, *Gm-csf*, *Il6*, *Vegf* and *Irf8* relative to WT littermates. At 18 weeks of age, *Ins2*^*Akita*/+^ retinas continued to show elevated levels of *Csf*, *Gm-csf*, *Il1β* and *Cd68.* Samples from 24-week-old *Ins2*^*Akita*/+^ mice showed reductions in mRNA, with levels close to or below baseline levels.

In comparison with the retinas from *Ins2*^*Akita*/+^ mice, those from *Lepr*^*db/db*^ mice showed marked differences in the mRNA levels of inflammatory markers (Fig. 1c). At both 3 and 24 weeks of age, many proinflammatory mRNAs appeared to be reduced relative to WT littermates. At 3 weeks of age, there were significantly decreased levels of *Csf*, *Vegf*, *Ccl5*, *Thbs1*, *Thbs2* and *Iba1.* There were also reductions in *Gm-csf*, *Ifnα*, *Ifnγ*, *Il1β*, *Il6*, *Cd68* and *Irf8* relative to WT littermates, although these were not significant. Similar to samples from 3-week-old mice, at 24 weeks of age, there were significantly decreased levels of *Csf*, *Gm-csf*, *Ifnα*, *Ifnγ*, *Vegf*, *Cd68*, *Thbs1*, *Irf8* and *Iba1.* At 6, 12 and 18 weeks of age, there were incremental increases in many of the mRNAs, with the peak appearing at around 18 weeks of age.

To examine potential changes in levels of proinflammatory factors at the protein level, multiplex ELISAs were analysed using whole retinal samples from 6- and 12-week-old diabetic *Ins2*^*Akita*/+^ and *Lepr*^*db/db*^ mice and WT mice (Fig. 2). In retinas from 6-week-old *Ins2*^*Akita*/+^ mice, there were significant increases in the levels of IFNγ, IL-1β and keratinocyte chemoattractant/growth-regulated oncogene (KC-GRO) compared with WT samples. In comparison, there were significant decreases in IL-5, IL-6 and IL-12p70 in retinas from 6-week-old *Lepr*^*db/db*^ mice compared with WT mice. Similar to mRNA levels, there was an overall decrease in the levels of proinflammatory factors found in the retinas of the *Lepr*^*db/db*^ mice; however, most findings did not reach statistical significance. At 12 weeks of age, IFN*γ* was the only factor that was significantly increased in the *Ins2*^*Akita*/+^ mice vs control littermates (Fig. 2). IFN*γ* was also the only proinflammatory factor that increased in *Lepr*^*db/db*^ mice in comparison with WT littermates, but the difference was not statistically significant.

**Fig. 3:**
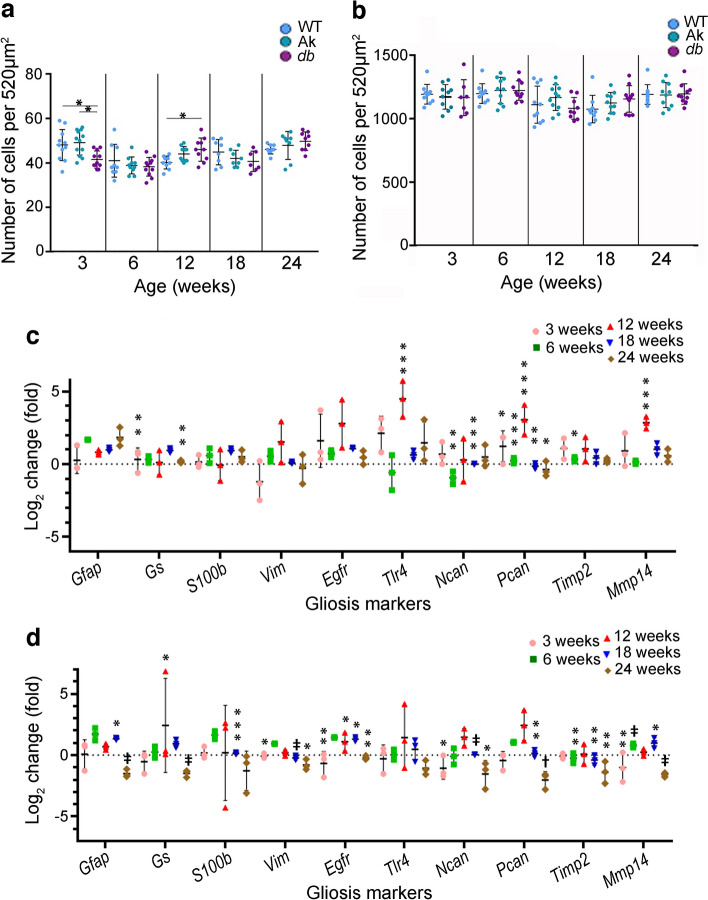
Retinal gliosis accompanies early diabetes in both *Ins2*^*Akita/+*^ (Ak) and *Lepr*^*db/db*^ (*db*) mice, with different rates of progression. (**a**, **b**) Murine retinal flatmounts from 3-, 6-, 12-, 18- and 24-week-old WT, Ak and *db* mice were immunolabelled with the myeloid lineage marker IBA1 (**a**) or the retinal astrocyte, Müller and cholinergic amacrine cell marker SOX2 (**b**), and the number of positive cells per 520 μm^2^ of tissue was quantified. Statistical analysis of data for IBA1^+^ (**a**) and SOX2^+^ (**b**) cells compared WT, Ak and *db* mice at each age. (**c**, **d**) Quantification of qRT-PCR data showing the log_2_ fold change in mRNA for factors that are altered during astrogliosis in Ak (**c**) and *db* (**d**) retinas relative to WT levels (WT levels were set to a value of 1.0) at 3, 6, 12, 18 and 24 weeks of age. Dotted line indicates a value of 0. Statistical analysis of RNA levels for each marker were compared for WT vs Ak (**c**) or WT vs *db* (**d**) across all ages. Overall, small changes in the number of IBA1^+^ cells were observed in retinas from *db* mice at 3 and 12 weeks of age in comparison with WT, but no differences in the number of retinal astrocytes were detected in the retinas of Ak or *db* mice in comparison with WT. Regarding statistically significant increases in gliosis markers (indicating the presence of gliosis), samples from 12-week-old Ak mice had an increase in *Tlr4*, *Pcan* and *Mmp14*. In samples from *db* mice, mRNA levels of *Gs* and *Egfr* were increased at 12 weeks of age, and *Gfap*, *Egfr* and *Mmp14* were increased at 18 weeks. Data are presented as mean±SEM; for (**a**) and (**b**), *n*=5–6 biological replicates, with three technical replicates for each biological replicate; for (**c**) and (**d**), *n*=3 biological replicates, with six technical replicates for each biological replicate. *p<0.05, **p<0.01, ***p<0.001, ^‡^*p*<0.0001; analysed by one-way ANOVA with Tukey post hoc analysis

Increases in proinflammatory chemokines and cytokines may be accompanied by changes to microglia numbers and/or infiltration of macrophages to the retina [23]. To determine whether there were changes to the number of cells of the myeloid lineage, retinal flatmounts from 3-, 6-, 12-, 18- and 24-week-old *Ins2*^*Akita*/+^ and *Lepr*^*db/db*^ mice were immunolabelled with an antibody recognising ionised calcium binding adaptor molecule 1 (IBA1; ESM Fig. 3). Analysis of cell counts from WT vs *Ins2*^*Akita*/+^ samples indicated there were no significant differences in the number of IBA1^+^ cells at any of the stages examined (Fig. 3a). In contrast, a small decrease in the number of IBA1^+^ cells was observed in *Lepr*^*db/db*^ mice at 3 weeks of age (41.6±1.2) vs WT mice (48.2±2.9) , while, at 12 weeks of age, there was a small increase in the mean number of IBA1^+^ cells in *Lepr*^*db/db*^ mice (46.0±1.6) vs WT mice (40.2±1.0). All other time points showed no differences in the number of IBA1^+^ cells in WT and *Lepr*^*db/db*^ mice.

### Gliosis was apparent in ***Ins2***^***Akita***/+^ and ***Lepr***^***db/db***^ samples between 6 and 18 weeks of age

Proinflammatory states are frequently accompanied by an activated state of astrocytes, termed gliosis, in which the cells de-differentiate, increase expression of growth factors and pro- and anti-inflammatory factors, and, in some cases, proliferate [25]. To determine whether there were changes to the number of astrocytes, retinal flatmounts from 3-, 6-, 12-, 18- and 24-week-old *Ins2*^*Akita*/+^ and *Lepr*^*db/db*^ mice were immunolabelled using antibodies specific for the transcription factor sex-determining region Y-box 2 (SOX2), a marker for astrocytes, cholinergic amacrine cells and progenitor cells [26]. In comparison with WT mice, retinas from *Ins2*^*Akita*/+^ and *Lepr*^*db/db*^ mice showed no statistically significant changes in the number of SOX2^+^ cells (Fig. 3b and ESM Fig. 4).

To determine the presence of gliosis, qRT-PCR was used to analyse mRNA levels of markers that are known to change during gliosis; analyses were carried out in retinal samples obtained from 3-, 6-, 12-, 18- and 24-week-old *Ins2*^*Akita*/+^ and *Lepr*^*db/db*^ mice [27, 28]. In 3-week-old *Ins2*^*Akita*/+^ mice, there was an increase in the level of *Ptprz1* (encoding phosphocan; also known as *Pcan*) in comparison with WT mice. In addition, *Gfap* (encoding glial fibrillary acidic protein) was elevated in the retinas of 6-week-old *Ins2*^*Akita*/+^ mice, although this difference was not significant. Similar to the results for mRNA levels of inflammatory markers, the peak in levels of mRNA associated with gliosis in *Ins2*^*Akita*/+^ mice appeared at 12 weeks of age, with increases in *Tlr4* (encoding TLR4), *Pcan* and *Mmp14* (encoding matrix metalloproteinase 14). Although some mRNA levels were still increased in samples from 18- and/or 24-week-old mice vs WT, the observed increases were not as great when compared with samples from 12-week-old mice.

In retinal samples from *Lepr*^*db/db*^ mice, there were some overall similarities between the data for mRNA levels of gliosis markers (Fig. 3d) vs inflammatory markers (Fig. 1), in that many of the mRNAs appeared to be unchanged or reduced in comparison with WT samples at 3 and 24 weeks of age, whereas multiple markers were increased in samples from 6-, 12- and 18-week-old *Lepr*^*db/db*^ mice (Fig. 3d). Gliosis markers that were significantly decreased in *Lepr*^*db/db*^ in comparison with samples from 24-week-old WT littermates included *Gfap*, *Glul* (encoding glutamine synthetase; also known as *Gs*), *Vim* (encoding vimentin), *Egfr* (encoding epidermal growth factor receptor), *Ncan* (encoding neurocan), *Pcan*, *Timp2* (encoding tissue inhibitor of metalloproteinase 2) and *Mmp14*. At 6 weeks of age, the retinas of *Lepr*^*db/db*^ mice had increased levels of *Gfap*, *S100b* (encoding S100 calcium-binding protein B), and *Egfr*, but these differences were not significant. At 12 weeks of age, there were increases in *Gs* and *Egfr* in *Lepr*^*db/db*^ vs WT, while at 18 weeks of age there were small increases in *Gfap*, *Egfr* and *Mmp14*, and decreases in *S100b*, *Vim*, *Ncan*, *Pcan* and *Timp2*. As with the retinal samples from *Ins2*^*Akita*/+^ mice, the peak increases in gliosis markers in *Lepr*^*db/db*^ mouse-derived retinas was observed at 12 weeks of age, specifically in *Gs* and *Egfr*, whereas levels for *Gfap*, *Egfr* and *Mmp14* were slightly increased at 18 weeks of age in comparison with WT samples.

### Retinal vasculature was altered at early timepoints in ***Ins2***^***Akita***/+^ and ***Lepr***^***db/db***^ models

**Fig. 4:**
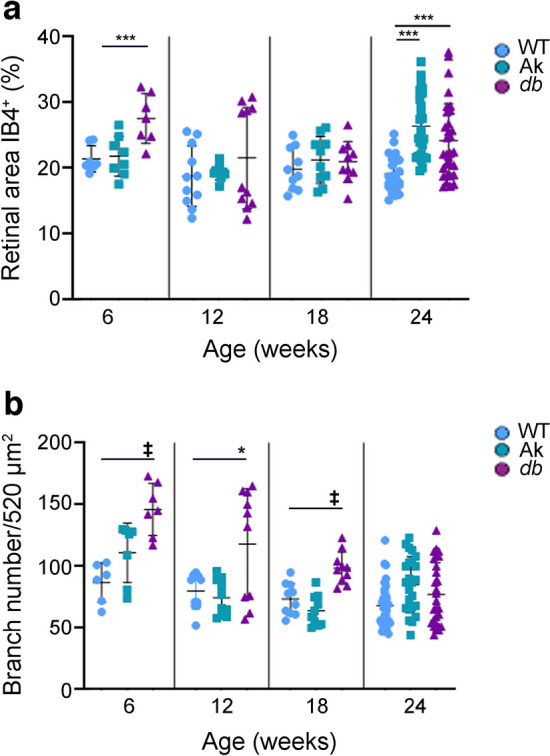
The percentage of the retina covered by IB4^+^ cells and number of branches were altered at various ages in *Ins2*^Akita/+^ (Ak) and *Lepr*^*db/db*^ (*db*) mice in comparison with WT mice. Flatmounts of retinas from 6-, 12-, 18- and 24-week-old WT, Ak and *db* mice were immunolabelled with the endothelial cell marker IB4, and digital images from the retinas were subjected to analysis using Angiotool software [30]. (**a**) Percentage of retinal area covered by IB4^+^ vasculature. (**b**) Number of branch points per 520 μm^2^ of tissue. Statistical analysis compared WT, Ak and *db* mice at each age. Retinal area covered by superficial plexus vasculature was increased in retinas from *db* mice at 6 weeks and in retinas of both Ak and *db* mice at 24 weeks in comparison with WT mice. The number of branches that were labelled with marker IB4 were increased at 6, 12 and 18 weeks of age in *db* mice, but no change in branch number was noted at any age in retinas of Ak mice in comparison with WT mice. Data are presented as mean±SEM; *n*=5–8 biological replicates, with six technical replicates for each biological replicate. *p<0.05, ***p<0.001, ^‡^*p*<0.0001, analysed by one-way ANOVA with Tukey post hoc analysis

Retinal microvascular abnormalities are commonly associated with diabetes, and include microaneurysms, haemorrhaging, intraretinal vascular abnormalities and neovascularisation in humans [29]. Retinas of *Ins2*^*Akita*/+^ and *Lepr*^*db/db*^ mice were immunolabelled with isolectin B4 (IB4) and the flatmounts of samples obtained at 6, 12, 18 and 24 weeks of age were analysed for percentage of retinal area covered by IB4^+^ cells and branch numbers using Angiotool software [30]. Retinas from *Ins2*^*Akita*/+^ mice showed no change in the percentage of retinal area covered by superficial vascular plexus until 24 weeks, at which time the percentage of retinal area covered by vasculature in *Lepr*^*db/db*^ samples was 25.80±1.0 in comparison with retinas of WT mice, in which 18.95±0.51 of retinal area was covered. In contrast, *Lepr*^*db/db*^ samples showed an increase at 6 weeks in comparison with WT and *Ins2*^*Akita*/+^ samples (Fig. 4a and ESM Fig. 5). The percentage area covered by IB4^+^ cells appeared to normalise in samples from 12- and 18-week-old mice but increased again at 24 weeks of age in comparison with WT mice. In *Lepr*^*db/db*^ samples, there was an increase in the percentage of area covered at 6 (27.16±2.27) and 24 weeks of age (23.68±1.02) in comparison with WT at the same ages, respectively (21.4±1.06 and 18.96±0.51). For branch number, the retinas of *Ins2*^*Akita*/+^ mice were similar to those of WT mice, with slight increases at 6 and 24 weeks of age (Fig. 4b). In contrast, the retinas of *Lepr*^*db/db*^ mice consistently showed an increase in branch number in comparison with WT at 6, 12 and 18 weeks of age.

### Treatment with IFNγ altered PDGFRβ signalling in pericytes

**Fig. 5:**
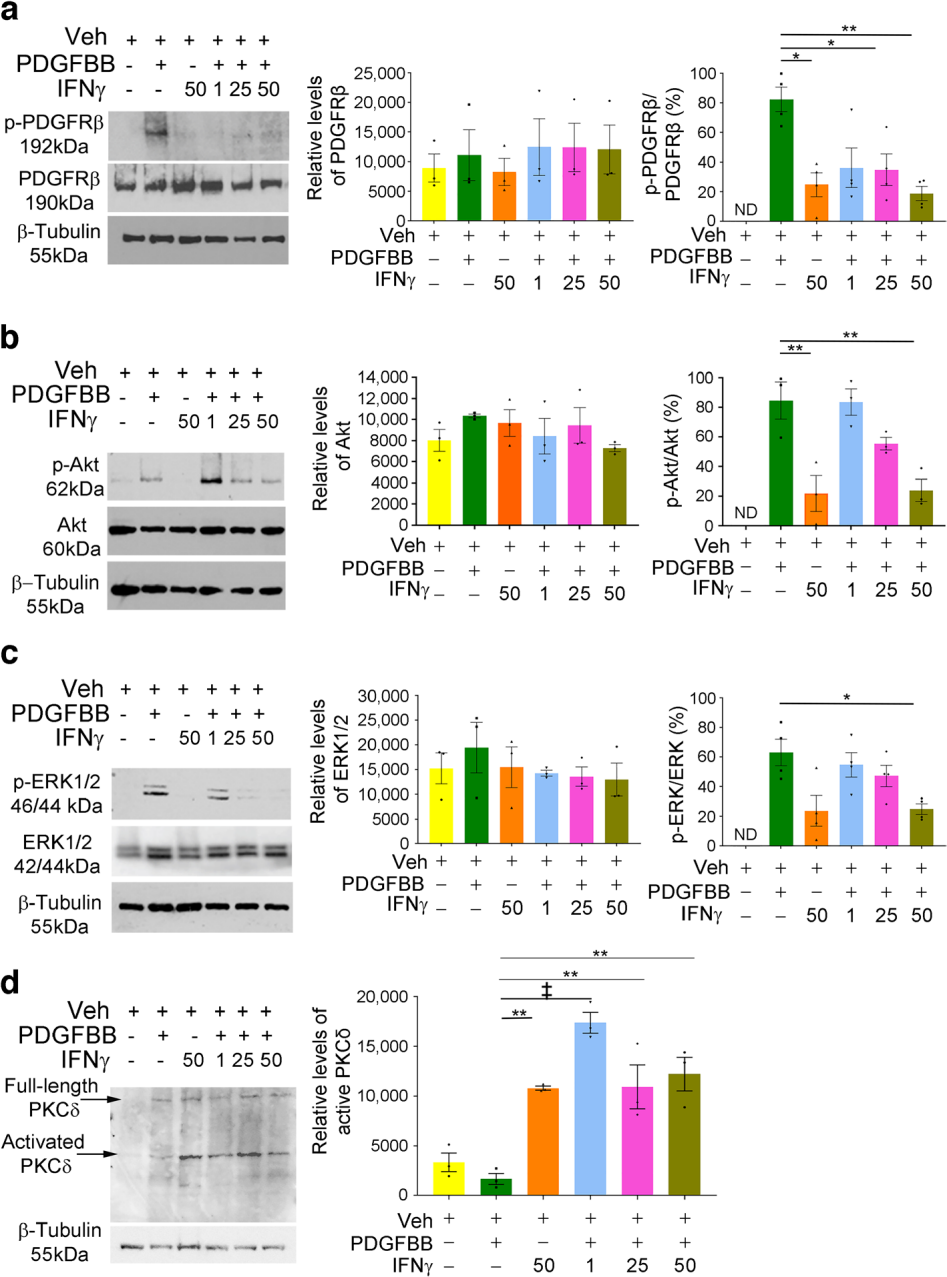
Chronic treatment of murine retinal pericytes with IFNγ reduced PDGFRβ, Akt and ERK signalling, and increased levels of cleaved PKCδ. (**a**–**d**) Lysates isolated from murine retinal pericytes incubated for 72 h with vehicle, PDGFBB alone, IFNγ alone, or 1 ng, 25 ng or 50 ng of IFNγ in the presence or absence of 50 ng PDGFBB were immunoblotted for p-PDGFRβ (Tyr751) and total PDGFRβ (**a**), p-Akt (Ser473) and total Akt (**b**), pERK1/2 (Thr202/Ty2204) and total ERK1/2 (**c**), or full-length and activated cleaved PKCδ (**d**). β-Tubulin and Revert Total Protein Stain were used to normalise readings from PKCδ blots, while β-tubulin was used to normalise the intensity readings of other blots. A representative blot for each label is shown, alongside densitometry data. The densitometry results show that, overall, treatment of isolated murine pericytes with increasing concentrations of IFNγ under chronic conditions decreased the levels of p-PDGFRβ, p-Akt and pERK1/2 as a ratio of the total amount of protein and increased the levels of cleaved PKCδ in comparison with vehicle and control treatments. ND, not detectable; Veh, vehicle. Data are presented as mean±SEM; for (**a**–**c**), *n*=3–4; for (**d**), *n*=3. *p<0.05, **p<0.01, ^‡^*p*<0.0001, analysed by one-way ANOVA with Tukey post hoc analysis

In the studies presented here, *IFNγ* levels were increased in both the *Ins2*^*Akita*/+^ and *Lepr*^*db/db*^ models at early stages of diabetes. To test the role of acute treatment with increasing concentrations of IFNγ in PDGFRβ signalling, isolated murine retinal pericytes were cultured and incubated with IFNγ for 24 h, incubated with PDGFBB for 20 min, and assayed for activation of PDGFRβ and downstream signalling pathways including PKB/Akt and mitogen-activated protein kinase (MAPK)/ERK1/2. No differences in PDGFRβ signalling were found between vehicle- and IFNγ-treated cells following 24 h of treatment (ESM Fig. 6). To determine whether longer exposure to IFNγ that mimicked chronic exposure perturbed PDGFRβ signalling, cells were treated with 1 ng, 25 ng or 50 ng of IFNγ for 72 h, followed by treatment with PDGFBB for 20 min. Lysates were again probed for PDGFRβ (Fig. 5a), Akt and p-Akt (Fig. 5b), or ERK1/2 and pERK1/2 (Fig. 5c). Densitometry readings normalised to β-tubulin indicated no changes in total levels of PDGFRβ, Akt or ERK1/2. Vehicle-treated samples showed no detectable level of p-PDGFRβ, and samples incubated with IFNγ alone showed a low level of p-PDGFRβ. In contrast, the ratio of normalised p-PDGFRβ to normalised total PDGFRβ had decreased in all IFNγ and PDGFBB co-treated samples in comparison with control samples (Fig. 5a). In addition, the ratios of p-Akt to total Akt and of pERK1/2 to total ERK1/2 showed statistically significant decreases in the 50 ng IFNγ+PDGFBB co-treatment conditions (Fig. 5b,c).

### Chronic IFNγ treatment induced PKCδ expression and reduced pericyte survival

An increase in the level of PKCδ has specifically been tied to pericyte loss in diabetic retinopathy [10]. To test the hypothesis that IFNγ may drive an increase in the levels of PKCδ, cultured pericytes were again incubated with 1 ng, 25 ng or 50 ng of IFNγ for 72 h in the presence of PDGFBB, and lysates were probed with an antibody that recognises the full-length and cleaved, activated forms of PKCδ (Fig. 5d). The mean densitometry levels of PKCδ were low in samples treated with vehicle or PDGFBB alone; however, relative levels increased significantly in samples treated with IFNγ alone and in the IFNγ+PDGFBB co-treated samples.

An increase in cleaved PKCδ suggests increased cell death associated with IFNγ treatment. To verify this, proliferation and cell death were directly assessed. Pericytes were grown in vehicle, 1 ng, 25 ng or 50 ng of IFNγ for 72 h in the presence of PDGFBB, and fixed cells were immunolabelled for cleaved caspase 3 (CC3) or proliferating cell nuclear antigen (PCNA). The number of positive cells for each marker was quantified as a percentage of the total number of cells (ESM Fig. 7). Vehicle-treated cells showed less than 1% of CC3^+^ cells (ESM Fig. 7a). In contrast, cells treated with IFNγ alone or with PDGFBB showed increasing numbers of CC3^+^ cells, concomitant with increasing concentrations of IFNγ (ESM Fig. 7a). No change in the percentage of PCNA^+^ cells was noted in IFNγ- and vehicle-treated pericytes (ESM Fig. 7b).

**Fig. 6:**
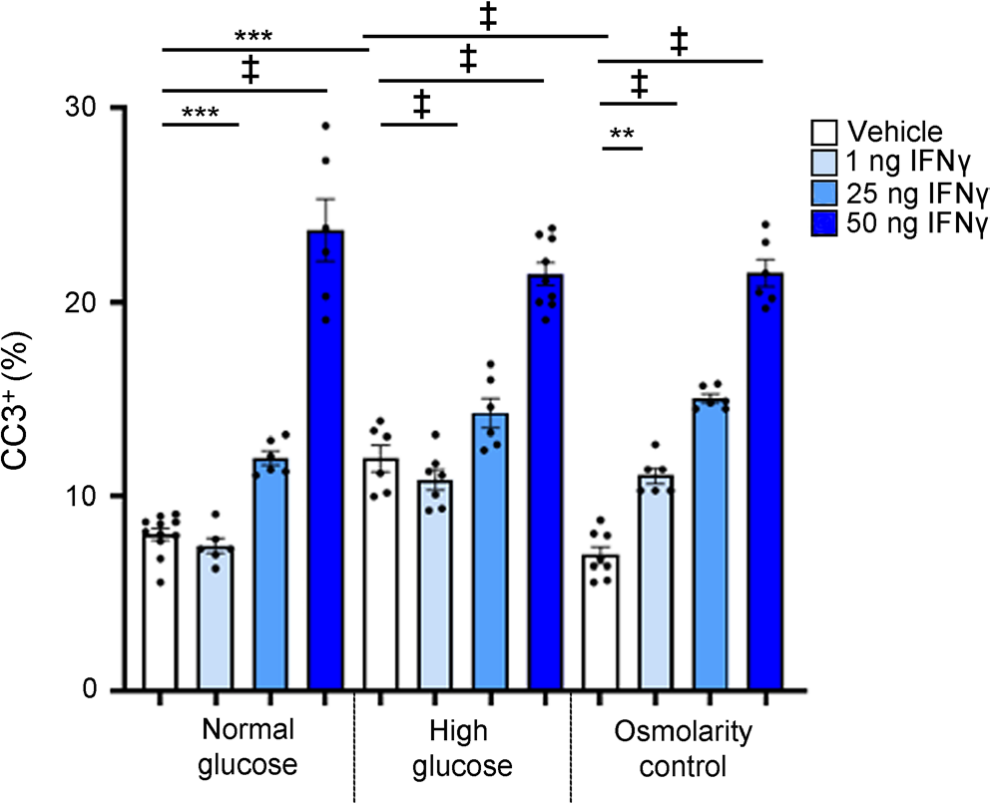
IFNγ increases pericyte death in media containing normal and high glucose levels. Numbers of CC3^+^ pericytes were quantified in media containing normal glucose levels (5.7 mmol/l d-glucose) or high glucose levels (40.7 mmol/l d-glucose), or in an osmolarity control (5.7 mmol/l d-glucose and 35mmol/l l-glucose). Cells were treated with vehicle, or 1 ng, 25 ng or 50 ng IFNγ. The number of CC3^+^ cells is expressed as a percentage of total cells labelled using Hoechst dye (DNA dye). Treating isolated murine pericytes with increasing concentrations of IFNγ increased the percentage of CC3^+^ cells in media containing normal glucose levels or high glucose levels, and in the osmolarity control in comparison with vehicle-treated cells. Under high-glucose conditions, vehicle-treated cells showed an increased percentage of CC3^+^ cells in comparison with vehicle-treated cells under normal glucose and osmolarity control conditions. Data are presented as mean±SEM;* n*=6–10. **p<0.01, ***p<0.001, ^‡^*p*<0.0001, analysed by one-way ANOVA with Tukey post hoc analysis

To investigate the role of IFNγ under hyperglycaemic conditions, pericytes were grown with vehicle or 1 ng, 25 ng or 50 ng of IFNγ in physiologically normal medium (5.7 mmol/l d-glucose), high-glucose medium (40.7 mmol/l d-glucose) or an osmotic control (35 mmol/l l-glucose with 5.7 mmol/l d-glucose), and the number of CC3^+^ pericytes was quantified as a percentage of total cells (Fig. 6). Under high-glucose conditions, vehicle-treated cells showed an increased percentage of CC3^+^ cells in comparison with vehicle-treated cells under normal glucose and osmolarity control conditions, while all cultures treated with 25 ng or 50 ng IFNγ showed an increase in percentage of CC3^+^ pericytes in comparison with those treated with vehicle or 1 ng IFNγ.

## Discussion

Levels of IFNγ are increased in individuals with diabetes with diabetic retinopathy, but not in those with diabetes without diabetic retinopathy, and are increased in both type 1 and type 2 diabetes [31]. The mechanisms by which IFNγ supports the development of diabetic retinopathy have not been investigated. Previous work from the Sheibani laboratory demonstrated that inflammation was the primary initiating factor in pericyte death under high-glucose conditions, and that Bcl-2 interacting mediator of cell death (BIM) and PKCδ were critical to pericyte death [18]. The study presented here extends those findings, and indicates that IFNγ is critical to the loss of pericytes and that the mechanism underlying this loss may, in part, involve a loss of PDGFRβ signalling.

Type 1 diabetes is an autoimmune disease in which multiple immune cells contribute to the accumulation of inflammatory molecules and the eventual destruction of the pancreatic beta cells [32]. In contrast, visceral white adipose tissue is the major source of inflammation in type 2 diabetes, as the tissue is infiltrated by macrophages and T and B cells [32]. The involvement of different inflammatory agents and the timeline on which they accumulate may indicate which organs become involved and the time point at which they become involved. This may partially explain why there appears to be a reduced inflammatory response at 6 and 24 weeks of age in the *Lepr*^*db/db*^ model. Evidence suggests that there are elevated levels of anti-inflammatory factors in various disease states, including type 2 diabetes, and that the levels of these factors fluctuate [33]. TNF-α and IL-1β have often been mentioned as playing a key role in both type 1 and type 2 diabetes, and IFNγ is another proinflammatory factor that is critical to the pathogenesis of both types of diabetes, but has not been extensively studied in diabetic retinopathy [34].

A comparative analysis between humans and rodents indicates that proinflammatory factors such as TNF-α, IL-1β and IL-6 are correlated with diabetic retinopathy. All three factors are very prominent in proliferative diabetic retinopathy in humans [35–38]. Vinores’ group investigated streptozotocin (STZ)-treated mice and *Ins2*^*Akita*/+^ mice with a TNF-α knockout, showing that there was a reduction in leucostasis by 1–2 weeks of diabetes, but TNF-α does not affect blood–retina barrier breakdown or CC3 levels until 3 months of development [39]. Regarding IL-6, 72 vitreal proteins were shown to be altered in mice treated with STZ as compared with controls; however, in 52 of the 72 proteins, these effects were mitigated in mice treated with an IL-6 inhibitor 10 weeks after STZ treatment, indicating the importance of IL-6 at the 10-week timepoint following diabetes induction [40]. In addition, several studies have quantified IL-1β levels in STZ-treated rats, showing that there was an increase in IL-1β at various times following STZ treatment [41, 42]. An in vitro investigation of IL-1β indicated that it may play a role in apoptosis of retinal endothelial cells [42].

Hyperglycaemia and chronic inflammation, arising in both type 1 and type 2 diabetes, are thought to initiate injury within the retinal neurovascular unit, including breakdown of the blood–retina barrier, vascular damage, increase in retinal inflammation, gliosis and neuronal loss. The byproducts of hyperglycaemia, AGEs, regulate inflammation by increasing NF-κB nuclear localisation and induction of IL-1β, TNF-α, macrophage inflammatory protein 2 (MIP2), IL-6 and IL-10 [43]. Increases in serum levels of TNF-α and IL-1β can induce adhesion of leucocytes to capillaries and lead to vascular leakiness, oedema and capillary non-perfusion [44]. IFNγ levels are increased in serum, tears, and the vitreous and aqueous humours of individuals with diabetes with retinopathy, but not in those with diabetes without retinopathy [31]. These findings have led to the proposal that IFNγ promotes and sustains chronic inflammation in diabetic retinopathy.

Evidence from our study indicates that IFNγ may also be important in mouse models of diabetic retinopathy. Specifically, we found that *Ifnγ* mRNA levels are increased in both the *Ins2*^*Akita*/+^ and *Lepr*^*db/db*^ models and IFNγ protein levels are increased in the *Ins2*^*Akita*/+^ model. In addition, we found that IFNγ reduces PDGFRβ signalling, subsequently leading to a loss of pericytes. The fact that IFNγ levels are not increased until after a reduction in pericytes may reflect: (1) a change in the number of pericytes that have completed differentiation due to a loss of PDGFRβ signalling; (2) IFNγ-initiated pericyte loss prior to the increase in retinal inflammation; or (3) that other factors, alone or in conjunction with IFNγ, are important in the initiation of pericyte loss. Future experiments will be important in distinguishing between these possibilities.

### Supplementary Information

Below is the link to the electronic supplementary material.

Supplementary file1 (PDF 2995 KB)

## Data Availability

All resources generated and/or analysed during the current study are included and available in the published article and online ESM files. All transgenic mouse lines used are commercially available.

## Abbreviations

AGE: Advanced glycated end-product
CC3: Cleaved caspase 3
IB4: Isolectin B4
IBA1: Ionised calcium binding adaptor molecule 1
NG2: Neural-glial antigen 2
PCNA: Proliferating cell nuclear antigen
PDGFB: Platelet-derived growth factor B
PDGFBB: Platelet-derived growth factor B homodimer
PDGFRβ: Platelet-derived growth factor receptor β
PKCδ: Protein kinase C isoform delta
qRT-PCR: Quantitative reverse transcription PCR
RAGE: Receptor for advanced glycated end-products
SOX2: Sex-determining region Y-box 2
STZ: Streptozotocin
SV40: Simian virus 40
TLR: Toll-like receptor
WT: Wild-type
