# Supplementary material for: Retinal inflammation in murine models of type 1 and type 2 diabetes with diabetic retinopathy
Source: Diabetologia. 2023 Sep 5;66(11):2170–85. doi: 10.1007/s00125-023-05995-4 (PMC10541343; doi:10.1007/s00125-023-05995-4)
Supplement: Supplementary file 1 — Supplementary file1 (PDF 2995 KB) [file 125_2023_5995_MOESM1_ESM.pdf]

ESM Figure 1

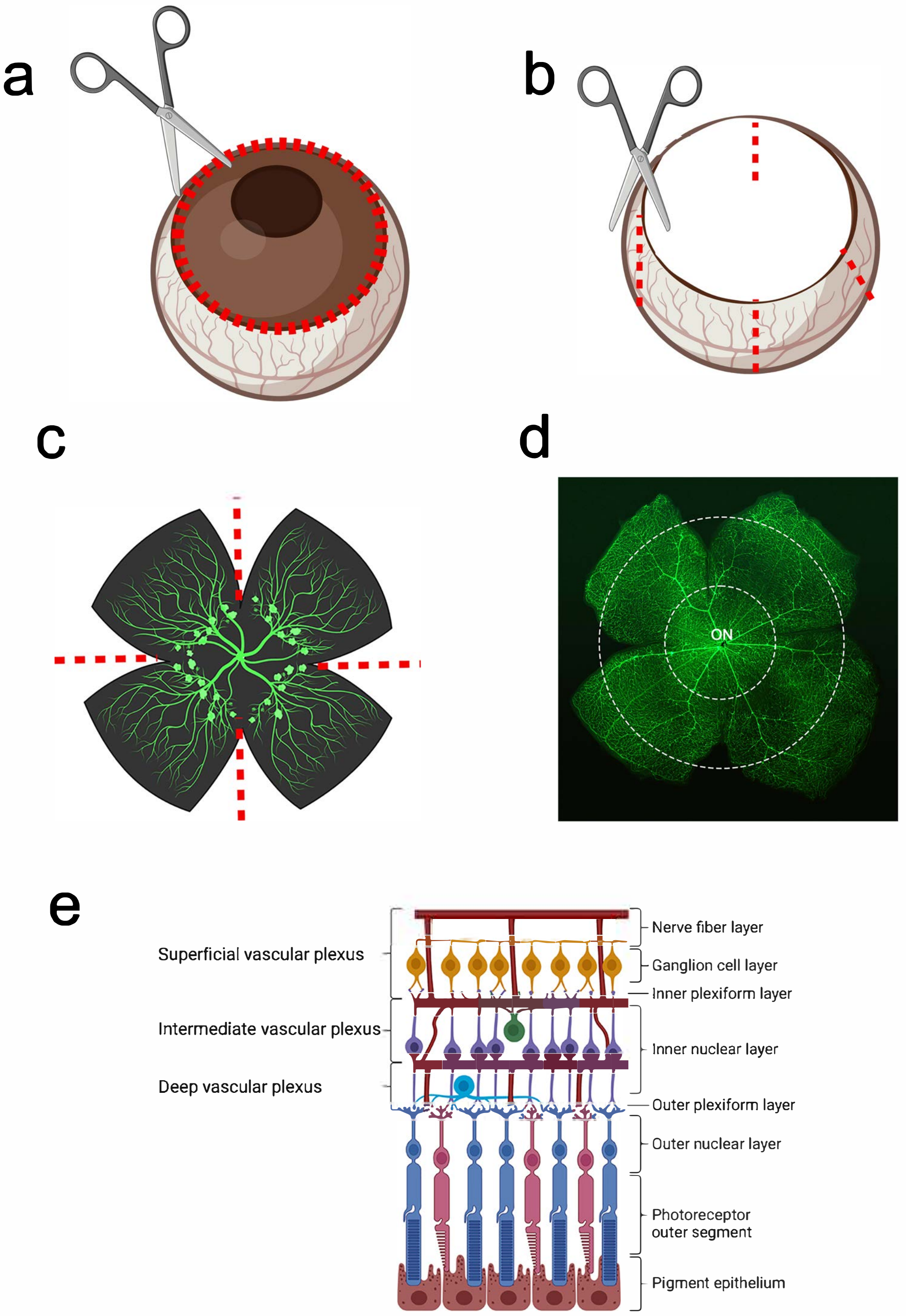

ESM Figure 1: Retinal flat mount and quantitation procedure. a) extraretinal tissues was removed from enucleated eyes. b) Cuts were placed in the retina to allow the retina to lie flat. c) Retinas were then labeled with isolectin B4 which labels vasculature (ESM Fig.5), or other cell type-specific markers such as NG2 (ESM Fig.2), IBA1 (ESM Fig.3), or SOX2 (ESM Fig.4).
d) Digital images were captured of 3-5 regions within 1-1000µm (inner circle) and more 1000µm away from the optic disc (outer circle) and the images were analyzed using FIJI image analysis (ESM Fig.2-4) or Angiotool software (ESM Fig.5). The region between the retina and the posterior hyaloid face which contains the superficial plexus and the cells contained therein.
e) The amount of retina and number of branches in the superficial vascular plexus was quantitated using Angiotool software.

ESM Figure 2

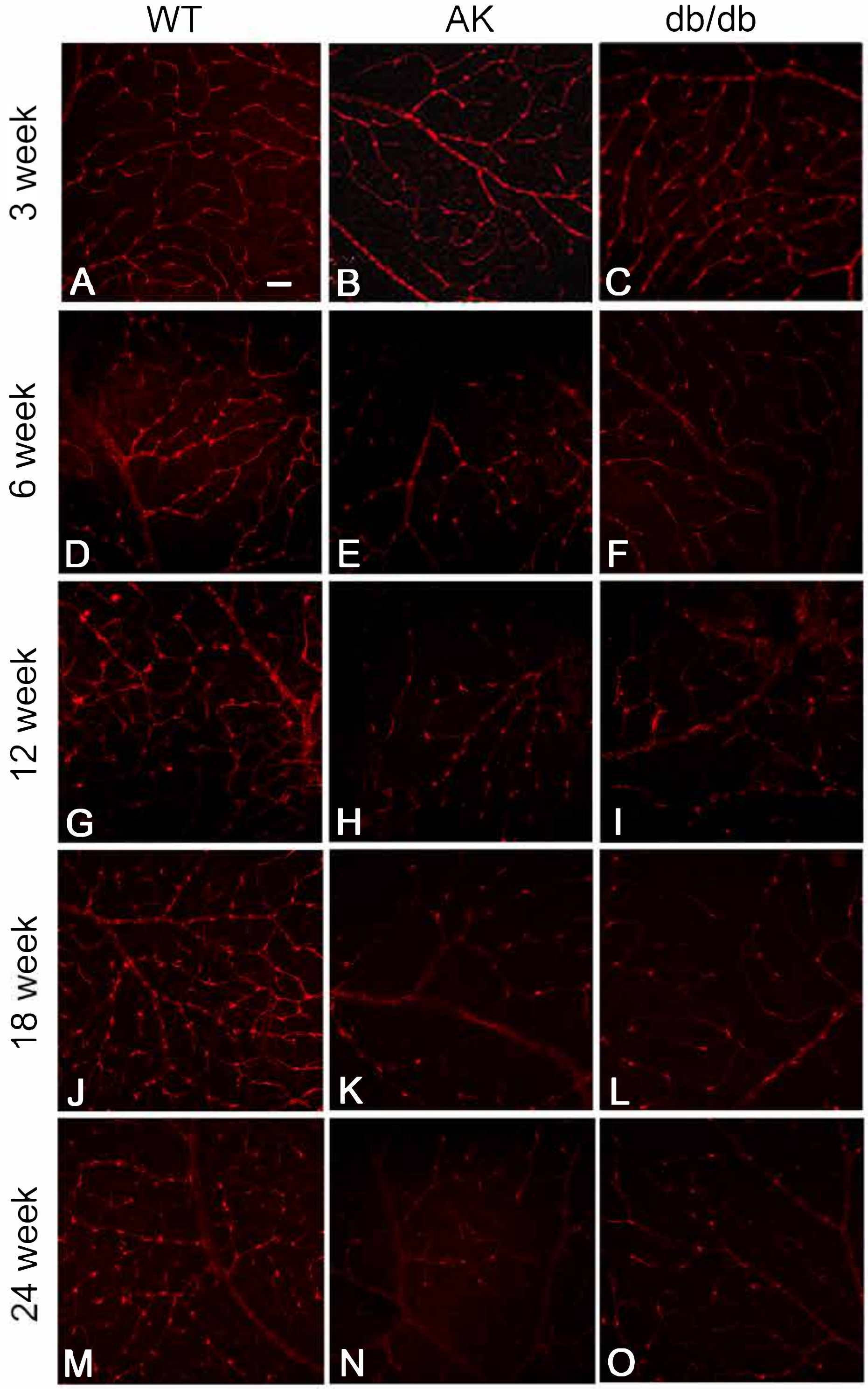

ESM Figure 2: NG2+ Cells in retinal flat mounts from WT, Ins2$^{Akita/+}$, and Lepr$^{db/db}$ retinas. Representative images were collected of retinal flat mounts from 3- (A-C), 6- (D-F), 12 -(G-I), 18- (J-L), and 24- week (M-O) WT (wt; A, D, G, J, M), Ins2$^{Akita/+}$ (Ak; B, E, H, K, N) and Lepr$^{db/db}$ (db/db; C, F, I, L,O) mice that had been immunolabeled for pericyte marker neural-glial antigen 2 (NG2). Images were used to quantitate the number of positive cells (See Fig.1a). Magnification bar in A=50 µm applies to A-O.

ESM Figure 3

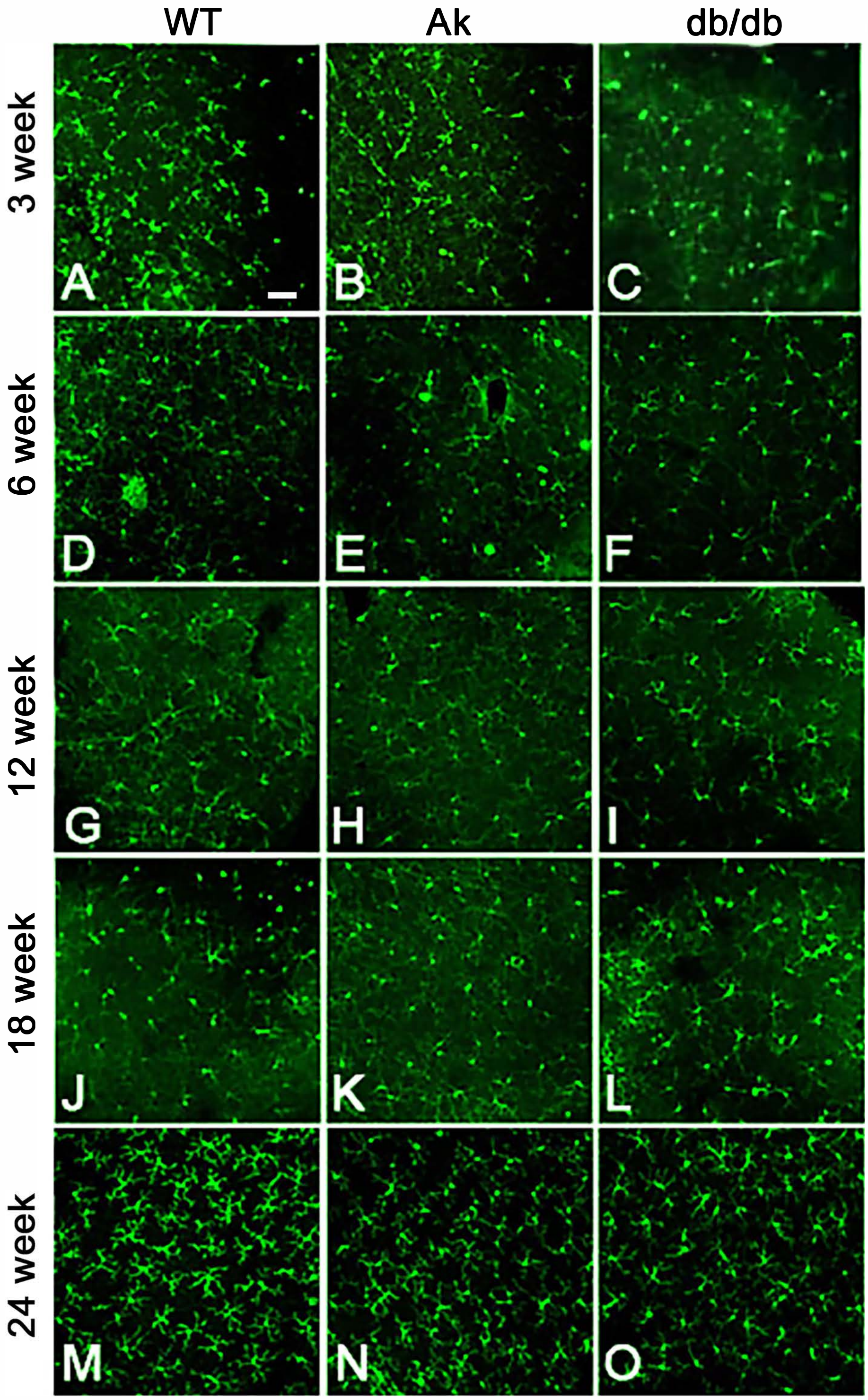

ESM Figure 3: IBA1+ cells in retinal flat mounts from WT, Ins2$^{Akita/+}$,and Lepr$^{db/db}$ retinas. Representative images were collected of retinal flat mounts from 3- (A-C), 6- (D-F), 12- (G-I), 18- (J-L),and 24-week (M-O) WT(A, D, G, J, M), Ins2$^{Akita/+}$ (Ak; B, E, H, K, N) and Lepr$^{db/db}$ (db/db; C, F, I, L, O) mice that had been immunolabeled for myeloid lineage marker (including microglia) ionized calcium binding adaptor molecule 1 (IBA1) and the number of positive cells quantitated (See Fig.2).

Magnification bar in A=50 µm applies to A-O.

ESM Figure 4

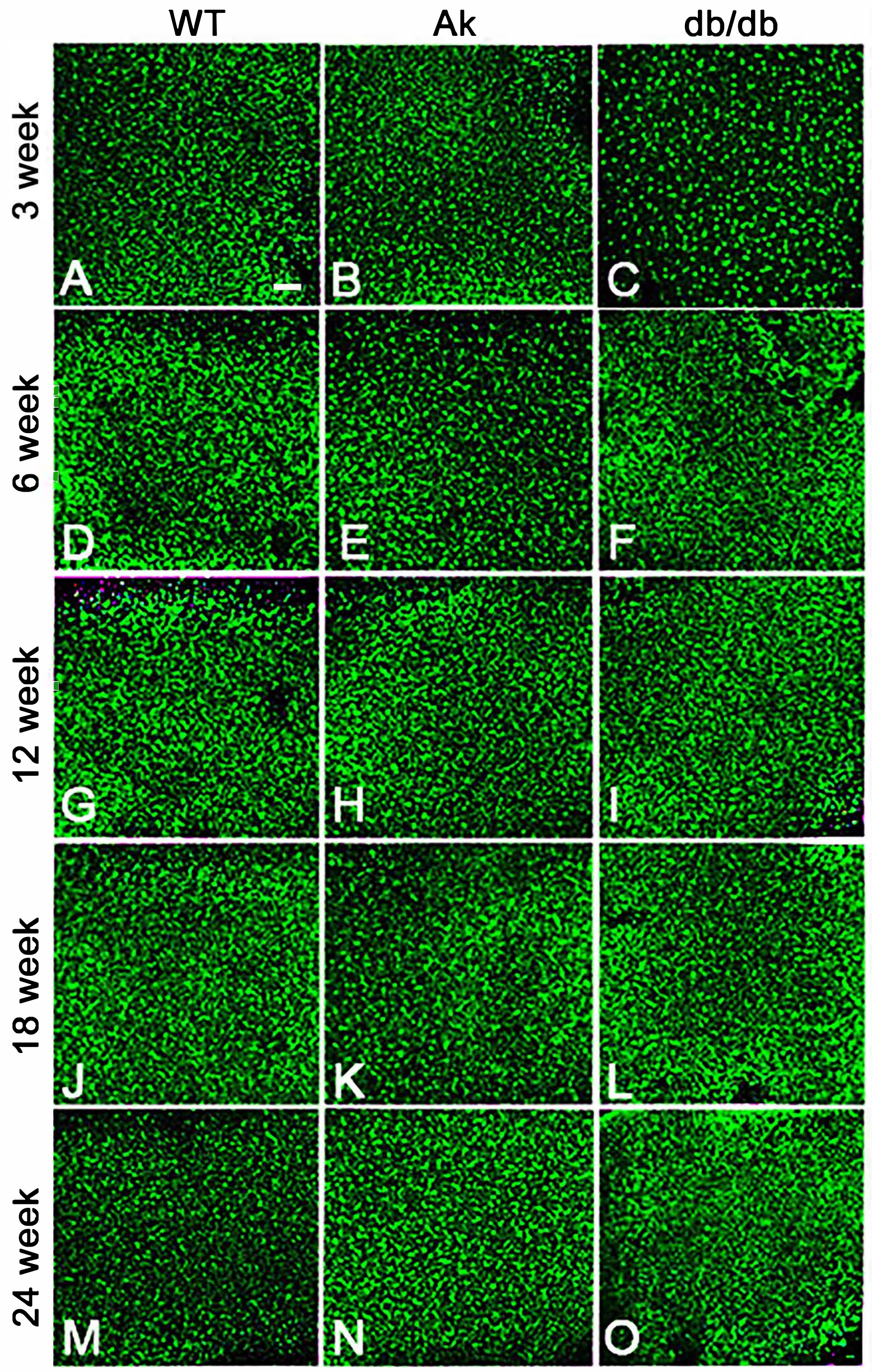

ESM Figure 4: SOX2+ cells in retinal flat mounts from WT, Ins2$^{Akita/+}$, and Lepr$^{db/db}$ retinas. Representative images were collected of retinal flat-mounts from 3- (A-C), 6- (D-F), 12- (G-I), 18- (J-L), and 24-week (M-O) WT (A, D, G, J, M), Ins2$^{Akita/+}$ (Ak; B, E, H, K, N) and Lepr$^{db/db}$ (db/db; C, F, I, L, O) mice that had been immunolabeled for astrocyte, Müller glial, and cholinergic amacrine cell marker sex determining region Y-box 2 (SOX2) and the number of positive cells quantitated (See Fig.2). Magnification bar in A=50 μm applies to A-O.

ESM Figure 5

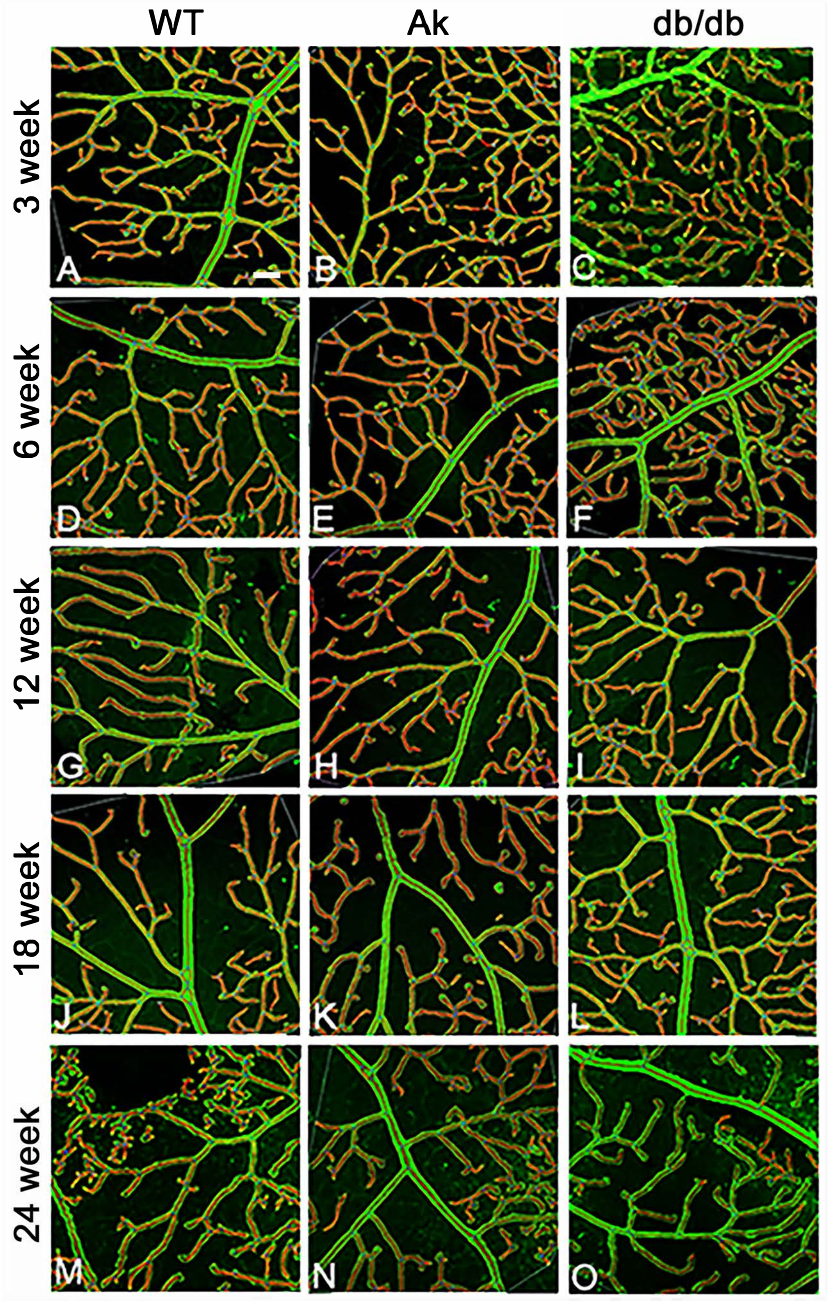

ESM Figure 5: IB4+ cells in retinal flat mounts from WT, Ins2Akita/+, and Leprdb/db retinas. Representative images were collected of retinal flat mounts from 3- (A-C), 6- (D-F), 12- (G-I), 18- (J-L), and 24-week (M-O) WT (A, D, G, J, M), Ins2Akita/+ (Ak; B, E, H, K, N) and Leprdb/db (db/db ; C, F, I, L, O) mice that had been labeled with endothelial cell marker Isolectin B-4 (IB4). Images were analyzed with Angiotool software to determine percentage of retinal area covered by vasculature and number of branch points (See Fig.4). Each figure shows IB4 label in green and Angiotool outline in red. Images were used to quantitate percentage of retinal area covered by vasculature and number of branch points graphed in Fig.4. Magnification bar in A=50 µm applies to A-O.

ESM Figure 6

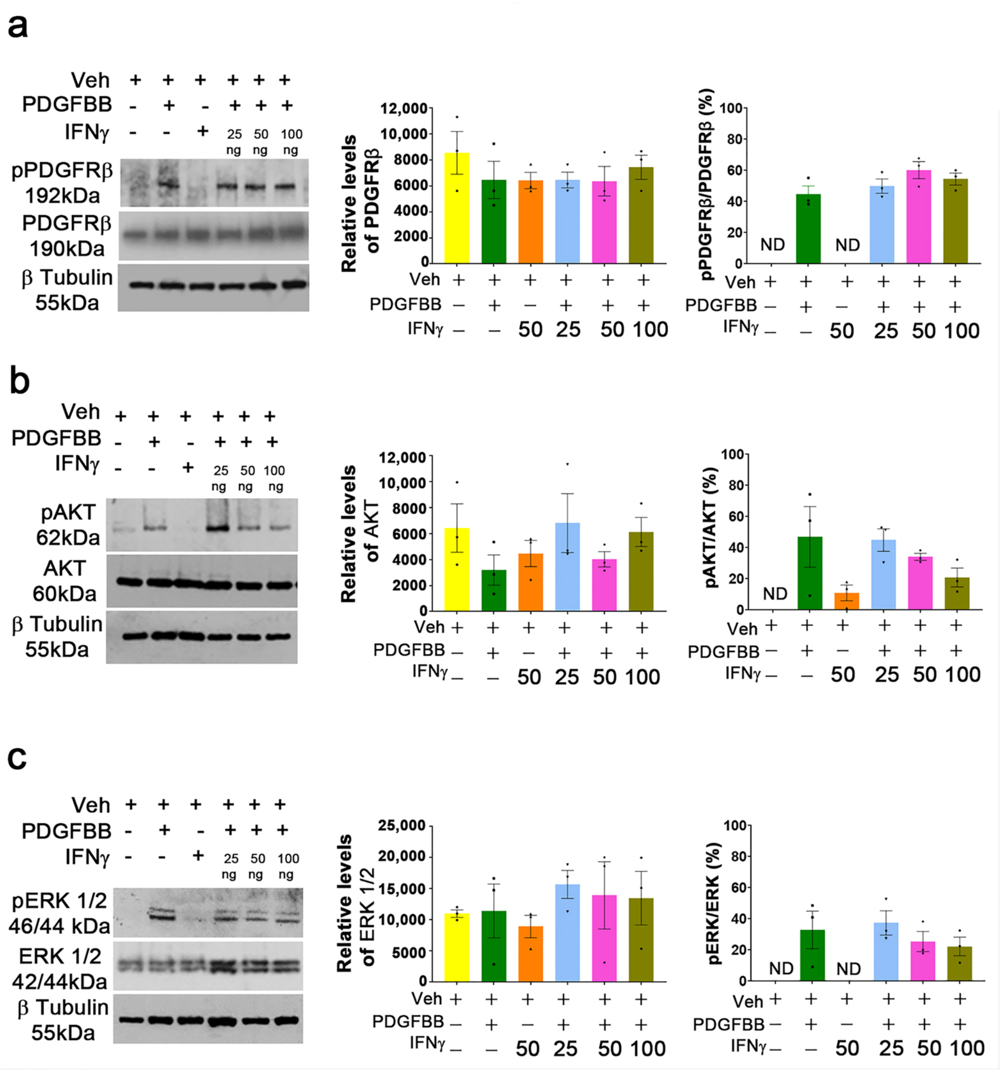

ESM Figure 6: Acute treatment of murine retinal pericytes with IFNγ did not alter PDGFRβ signaling. a, b, c) Lysates prepared from murine retinal pericytes incubated for 24 hours with Vehicle (Veh), PDGFBB alone, IFNγ alone, or 25, 50, or 100 ng of IFNγ with PDGFBB were immunoblotted for pPDGFRβ (Tyr751) and total PDGFRβ (a), pAKT (Ser473) and total AKT (b), or pERK 1/2 (Thr202/Tyr204) and total ERK 1/2 (c). Each label intensity was normalized using β-tubulin. A representative blot for each label is shown on the left-hand side of the figure, while densitometry from blots is shown on the right. Error bars represent SEM. Statistical significance was determined using a one-way ANOVA with Tukey post-hoc analysis (n=3-5). ND: not detectable.

ESM Figure 7

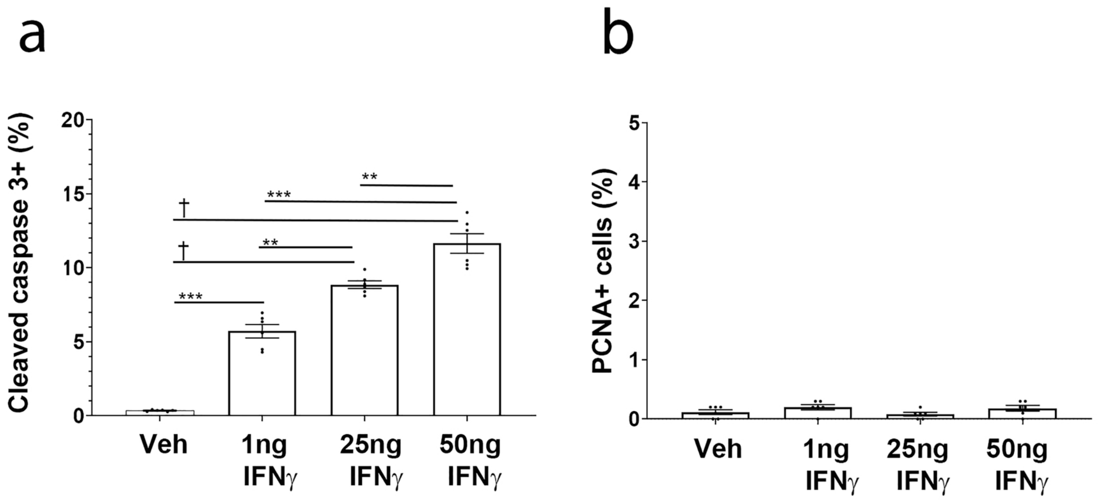

ESM Figure 7: IFNγ treatment increased the number of cleaved caspase 3-labeled murine retinal pericytes in vitro. Murine retinal pericytes were incubated for 72 hours with vehicle (Veh) or 1, 25, or 50 ng of IFNγ in the presence of 50 ng PDGFBB. Following treatment, cells were fixed and immunolabeled for cleaved caspase 3 or proliferating cell nuclear antigen and Hoechst for nuclei. Numbers of cleaved caspase 3+ (a) or proliferating cell nuclear antigen+ (PCNA+) (b) cells were quantitated. N=3 for each marker and condition. Error bars represent SEM. Statistical significance was determined using a one-way ANOVA with Tukey post-hoc analysis.
**$p<0.01$, †$p<0.001$, ‡$p<0.0001$.
